# Genotypic Shift and Diversification of MRSA Blood Stream Isolates in a University Hospital Setting: Evidence from a 12-Year Observational Study

**DOI:** 10.3390/antibiotics13070670

**Published:** 2024-07-19

**Authors:** Yuka Motomura, Motoyasu Miyazaki, Mitsuhiro Kamada, Shinichi Morimoto, Yoshihiko Nakamura, Tomomitsu Satho, Tohru Takata, Nobuhiro Kashige

**Affiliations:** 1 Faculty of Pharmaceutical Sciences, Fukuoka University, Fukuoka 814-0180, Japan; motomura@fihes.pref.fukuoka.jp (Y.M.); motoyasu@fukuoka-u.ac.jp (M.M.); satho@fukuoka-u.ac.jp (T.S.); kashige@fukuoka-u.ac.jp (N.K.); 2 Department of Pharmacy, Fukuoka University Chikushi Hospital, Fukuoka 818-8502, Japan; 3 Department of Pharmacy, Fukuoka University Hospital, Fukuoka 814-0180, Japan; mkamada@fukuoka-u.ac.jp; 4 Department of Emergency and Critical Care Medicine, Faculty of Medicine, Fukuoka University, Fukuoka 814-0180, Japan; otomirom7960@adm.fukuoka-u.ac.jp (S.M.); nakamura58@adm.fukuoka-u.ac.jp (Y.N.); 5 Department of Oncology, Hematology, and Infectious Diseases, Fukuoka University Hospital, Fukuoka 814-0180, Japan; 6 Department of Infection Control, Fukuoka University Hospital, Fukuoka 814-0180, Japan

**Keywords:** MRSA, BSI, SCC*mec*, *agr*, POT, MLST

## Abstract

There have been few reports regarding the long-term trends in the genotypes of methicillin-resistant *Staphylococcus aureus* (MRSA) bloodstream isolates. Therefore, this study was performed to investigate the longitudinal trends in the genotypes of MRSA bloodstream isolates obtained from hospitalized patients during a 12-year study period from 2010 to 2021 at a tertiary care university hospital. Over the 12-year period from 2010 to 2021, we conducted a genetic investigation focusing on 245 MRSA strains isolated from the blood of hospitalized patients. The genotypes of the MRSA bloodstream isolates were determined by Staphylococcal Cassette Chromosome *mec* (SCC*mec*) typing, accessory gene regulator (*agr*) typing, PCR-based ORF typing (POT), and multilocus sequence typing (MLST). Strains with the same POT type detected in two or more isolates were designated as epidemic clones, while strains without a common POT type were classified as sporadic clones. Until 2015, isolates with SCC*mec* II/*agr* II were prevalent, but isolates with SCC*mec* IV/*agr* III increased from 2016. A total of 128 strains (52%) were identified as epidemic clones, while 117 strains (48%) were classified as sporadic clones. The detection rate of sporadic clones increased significantly since 2016 (*p* < 0.05). The epidemic clones were classified into three clusters, with MRSA of clonal complex (CC) 1 being prominent after 2016. This study showed that the genotypes of MRSA bloodstream isolates underwent a shift from SCC*mec* II/*agr* II type to SCC*mec* IV/*agr* III type, with a notable increase in MRSA of CC1, after 2016. There was a significant increase in the proportion of sporadic strains among the isolates, suggesting the diversification of genotypes.

## 1. Introduction

The drug-resistant bacterium methicillin-resistant *Staphylococcus aureus* (MRSA) is a major pathogen in nosocomial infections in healthcare facilities. Bloodstream infections with MRSA, such as bacteremia and infective endocarditis, are severe conditions, constituting critical illnesses with high mortality rates [1].

From the late 1970s to the late 1980s, MRSA in Japanese healthcare facilities was primarily associated with the detection of Staphylococcal Cassette Chromosome *mec* (SCC*mec*) types I and IV [2,3]. However, from around the 1990s to the early 2000s, a multidrug-resistant MRSA strain characterized by the sequence type (ST)5-SCC*mec* type II became widespread in countries such as the USA, South Korea, and Japan [4,5,6].

Since the late 1990s, reports of fatalities resulting from community-acquired (CA)-MRSA infections have emerged [7], focusing attention on CA-MRSA characterized by SCC*mec* type IV [8,9]. In addition, from the 2000s, the USA300 strain carrying high-pathogenicity factors, such as Panton–Valentine leukocidin (PVL) and arginine catabolic mobile element (ACME), and exhibiting SCC*mec* type IV, which had been prevalent in the community, was also detected in healthcare facilities, particularly in the USA [10,11]. This strain led to severe infections, including necrotizing fasciitis [12].

In Japanese healthcare facilities, SCC*mec* type IV MRSA has been increasing in both non-blood isolates and blood isolates since the mid-2010s, indicating a shift in genotypic pattern [13,14,15,16]. With the increasing detection of CA-MRSA strains in healthcare settings, it has become important to analyze the longitudinal trends in the genotypes of MRSA from the perspectives of infection control and antimicrobial therapy.

The investigation of genotypes in MRSA blood isolates has been reported both domestically in Japan and internationally, but these studies focused predominantly on analyzing SCC*mec* types and sequence types in multilocus sequence typing (MLST) analysis [17,18,19,20,21,22,23,24,25,26,27,28]. There have been very few reports comprehensively examining longitudinal trends over periods of more than a decade for multiple genotypes, including SCC*mec* and accessory gene regulator (*agr*) typing. Previously, we investigated the genotypes of 137 MRSA bloodstream isolates from Fukuoka University Hospital over the 18-year period from 1987 to 2004 [29]. We reported a transition from SCC*mec* type IV/accessory gene regulator (*agr*) type III to SCC*mec* type II/*agr* type II during this period. The present study was performed to elucidate the longitudinal molecular epidemiological changes in MRSA blood isolates detected at our institution from 2010 to 2021. We conducted investigations using molecular epidemiological analysis methods, such as SCC*mec* typing, *agr* typing, PCR-based ORF typing (POT), and MLST. In addition, we explored the associations between antibiotic susceptibility patterns and various genotypes.

## 2. Results

### 2.1. Trends in SCCmec Types

**Figure 1:**
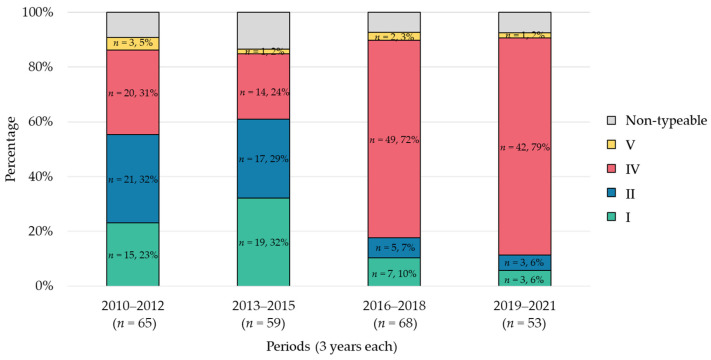
**Trends of changes in SCC*mec* types.** The data are presented as percentages relative to the total isolates for each period. The figure illustrates the distribution of SCC*mec* types across the four periods.

The trends of changes in SCC*mec* types over the years during the study period are shown in Figure 1. In the first period, type II (32%) was the most prevalent, followed by type IV (31%) and type I (23%). In the second period, type I (32%) was predominant, followed by type II (29%) and type IV (24%). However, type IV exhibited a significant increasing trend in prevalence from the third period (*p* < 0.01). In the fourth period, type IV (79%) was detected most frequently, followed by type I (6%) and type II (6%).

### 2.2. Trends in Agr Types

**Figure 2:**
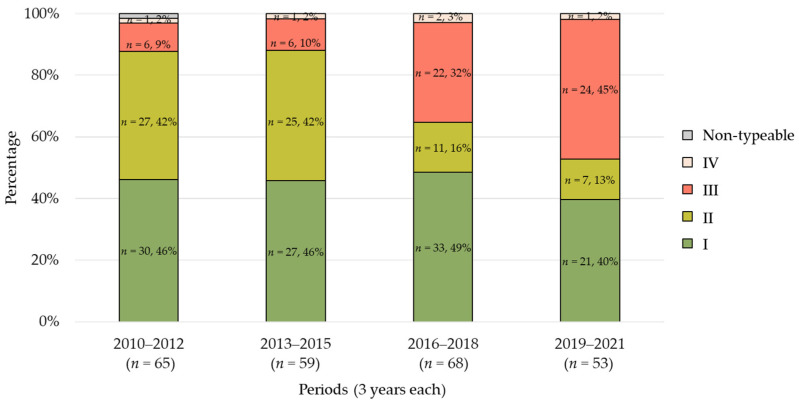
**Trends in *agr* types.** The data are presented as percentages relative to the total isolates for each period. The figure illustrates the distribution of *agr* types across the four periods.

The trends of changes in *agr* types over the years are shown in Figure 2. In both the first and second periods, type I (both 46%) was detected most frequently, followed by type II (both 42%) and type III (9% and 10%, respectively). However, type III showed a significant increasing trend from the third period (*p* < 0.01). In the fourth period, type III (45%) was the most prevalent, followed by type I (40%) and type II (13%), establishing the dominance of type III.

### 2.3. POT Type Distribution

**Figure 3:**
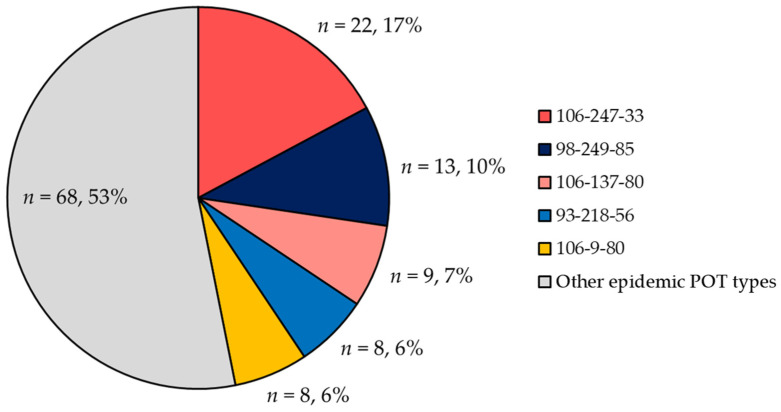
**Distribution of POT types among epidemic clones.** The distribution of the 128 isolates identified as epidemic clones is based on POT types. The total percentage is 99% as each component ratio is rounded to the nearest whole number.

Among the 245 MRSA bloodstream isolates, there were 143 distinct POT types, with 26 identified as epidemic clones (*n* = 128, 52%) and 117 as sporadic clones (48%). Among the epidemic clones, the top five POT types accounted for 45% (Figure 3). The genotypes (SCC*mec*/*agr*) of these POT types were as follows. POT type 106-247-33 (*n* = 22) were all identified as SCC*mec* type IV/*agr* type III. POT type 98-249-85 (*n* = 13) except for one isolate were SCC*mec* type I/*agr* type I. POT types 106-137-80 (*n* = 9) and 106-9-80 (*n* = 8) were all identified as SCC*mec* type IV/*agr* type I. POT type 93-218-56 (*n* = 8) were all SCC*mec* type II/*agr* type II (Table S1).

### 2.4. Trends in POT Types

**Figure 4:**
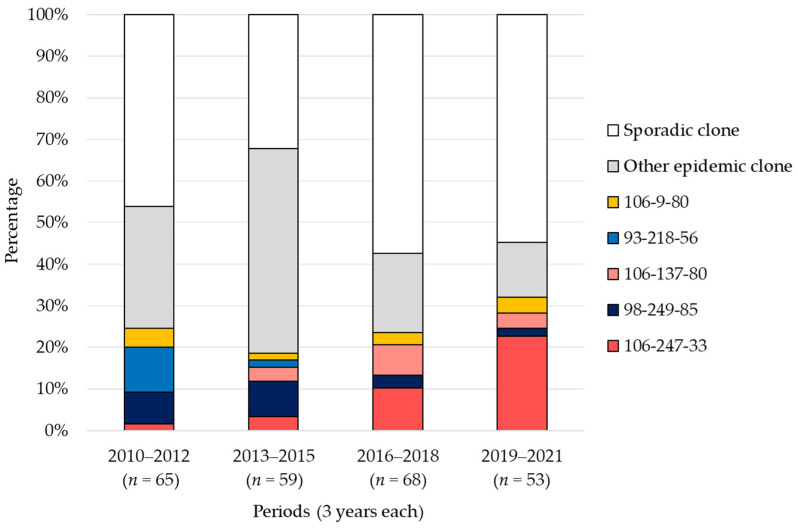
**Trends in major epidemic clones according to POT analysis.** The data are presented as percentages of total isolates for each period. The figure illustrates the longitudinal trends of the top 5 major epidemic clones classified by POT type, along with other epidemic clones and sporadic clones, for each of the four periods.

Figure 4 presents the longitudinal trends of the top five major epidemic clones classified by POT type, along with other epidemic clones and sporadic clones. POT type 93-218-56, characterized by SCC*mec* type II/*agr* type II, was detected at a rate of 11% in the first period and was a major strain. However, in the second period, the detection rate dropped to 2%, and it was not detected in the third and fourth periods. On the other hand, POT type 106-247-33, characterized by SCC*mec* type IV/*agr* type III, had detection rates of 2% and 3% in the first and second periods, respectively. However, it increased to 10% in the third period and rose further to 23% in the fourth period.

The detection rate of sporadic clones increased significantly in the third and fourth periods compared to the first and second periods [38% (49/124) vs. 56% (68/121), respectively; *p* = 0.013].

### 2.5. Cluster Analysis

**Figure 5:**
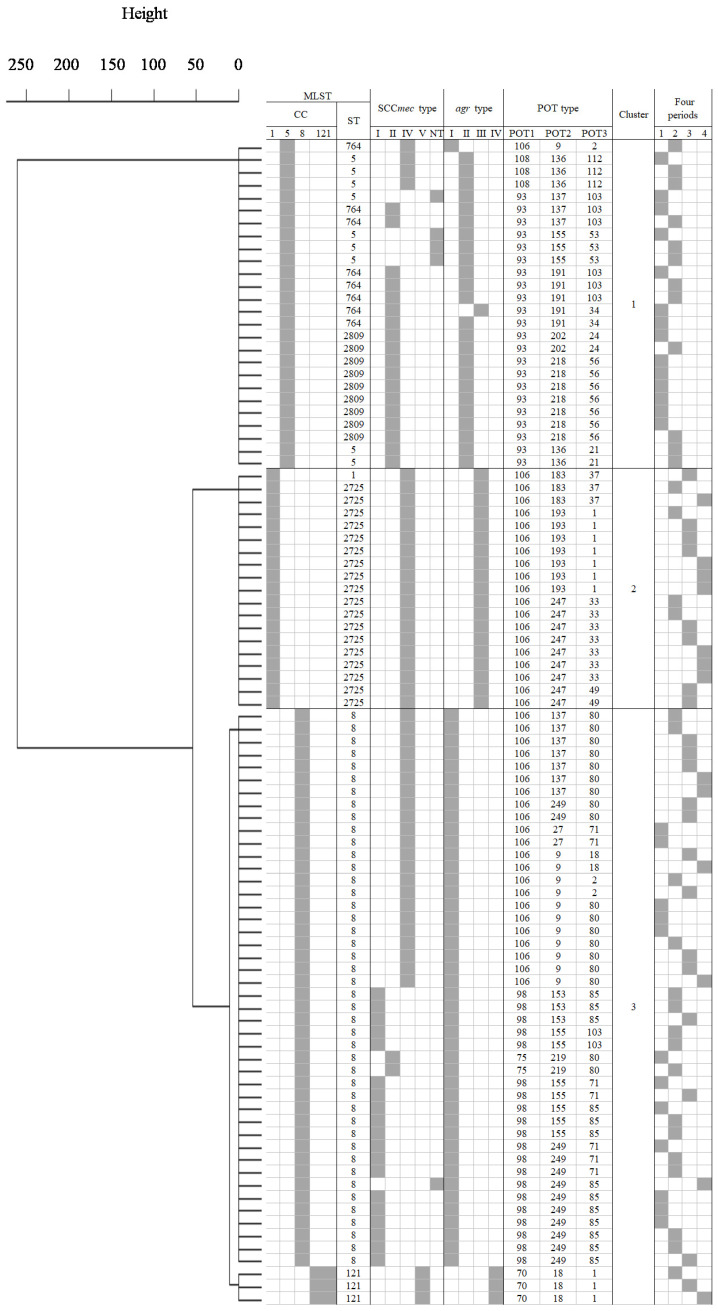
**Dendrogram and molecular epidemiological analysis of 92 randomly selected epidemic clones.** First column: CC analyzed by MLST. Second column: ST analyzed by MLST. Third column: SCC*mec* typing classification. Fourth column: *agr* typing classification. Fifth column: POT number. Sixth column: Cluster number. Seventh column: Periods of detection (first period: 2010–2012; second period: 2013–2015; third period: 2016–2018; and fourth period: 2019–2021, respectively).

Among the epidemic clones identified by POT analysis, we randomly selected samples with detection rates ranging from 32% (7/22) to 100% (2/2), totaling 92 strains, for MLST analysis. Figure 5 illustrates the results of cluster analysis based on MLST and POT analyses. The genotypes of epidemic strains showed three clusters with dissimilarity set at 50 (Cluster 1, 26 strains; Cluster 2, 19 strains; Cluster 3, 47 strains). Cluster 1 consisted of strains belonging to CC5, identified as ST2809, ST5, and ST764 and detected only in the first and second periods. The predominant SCC*mec* type was II (20/26), and the *agr* type was mainly classified as type II (24/26) within this cluster. Cluster 2 comprised all strains belonging to CC1, with ST identified as ST2725 for all except one isolate. This cluster was primarily detected in the third and fourth periods, and all strains within this cluster were characterized by SCC*mec* type IV and *agr* type III. Strains in Cluster 3 were identified as CC8, ST8 (*n* = 44) and CC121, ST121 (*n* = 3). ST8 was primarily classified into SCC*mec* type I (*n* = 19) and SCC*mec* type IV (*n* = 22), with all strains having *agr* type I. In the first and second periods, ST8 strains with SCC*mec* type I (*n* = 16) were predominantly detected, while in the third and fourth periods, strains with SCC*mec* type IV (*n* = 13) were mainly detected. ST121 strains were all classified as SCC*mec* type V and *agr* type IV, with one strain detected in each of the second, third, and fourth periods. The major five POT types had the following STs: 106-247-33 was ST2725; 98-249-85, 106-137-80, and 106-9-80 were ST8; and 93-218-56 was ST2809 (Table S1).

### 2.6. Identification of Virulence Genes and Antimicrobial Resistance Profiles

PVL (*lukS-PV*-*lukF-PV*) and ACME (*arc*, *opp3*) were detected for only one isolate in 2020. The POT type of this strain was identified as 106-77-113, which was the same as USA300. The SCCmec type was not identified (*ccr* gene: none, *mec* gene: classB), while *agr* was type I and ST was ST8 (Table S1: No. 222).

### 2.7. Antimicrobial Resistance Profiles

Table 1 presents the distributions of the mode of minimum inhibitory concentration (MIC) and resistance rates of antimicrobials over the study period for 244 MRSA blood isolates. All isolates showed susceptibility to both teicoplanin (TEIC) and linezolid (LZD), while one strain exhibited intermediate resistance to vancomycin (VCM) (4 μg/mL). Seven strains (4%) showed no susceptibility to daptomycin (DAP). The resistance rates for the other antimicrobials examined were as follows: arbekacin (ABK), 1% (*n* = 2); gentamicin (GM), 49% (*n* = 119); erythromycin (EM), 72% (*n* = 175); clindamycin (CLDM), 45% (*n* = 110); minocycline (MINO), 34% (*n* = 83); levofloxacin (LVFX), 79% (*n* = 192); and fosfomycin (FOM), 19% (*n* = 46). Over the 12-year study period, decreasing trends were observed in the detection rates of strains resistant to GM, EM, CLDM, MINO, and FOM (all *p* < 0.05).

### 2.8. Relations between Each Genotype (SCCmec and Agr Types) and VCM Susceptibility

A total of 14 strains with VCM MICs of 2 and 4 μg/mL were detected (Table S1, No.19, 55, 63, 66, 74, 81, 91, 118, 152, 160, 180, 207, 231). In the first and second periods, nine strains with VCM MICs of 2 or 4 μg/mL were detected, of which five strains were identified as SCC*mec* type II/*agr* type II. On the other hand, in the third and later periods, five strains with VCM MICs of 2 μg/mL were detected, of which three were identified as SCC*mec* type IV/*agr* type I and two as SCC*mec* type II/*agr* type II.

Strains with *agr* type II included a significantly higher proportion of strains with VCM MICs of 2 or 4 μg/mL compared to strains with other *agr* types [13% (9/70) vs. 3% (5/174), respectively; *p* < 0.01].

## 3. Discussion

This study was performed to investigate the longitudinal trends in the genotypes of MRSA bloodstream isolates over a period of 12 years. Taken together with previous reports, the duration of monitoring spans over 30 years. Studies investigating the epidemiological trends of bloodstream isolates over such an extended period are exceedingly rare.

In our hospital, the SCC*mec* types of MRSA bloodstream isolates have undergone a significant transition from types I and II to type IV since 2016 with corresponding changes in drug susceptibility. Similar trends were observed in changes in SCC*mec* types for non-blood and blood isolates in other healthcare facilities in Japan [14,16,21]. Interestingly, SCC*mec* type IV, known to be associated with CA-MRSA, was sporadically detected in healthcare facilities in Japan, including our hospital, from the early 1970s to the late 1980s [2,29].

Similar to SCC*mec* types, the *agr* type also underwent a significant transition from type II to type III after 2016. While it has been reported that *agr* type II is predominant in hospital-acquired (HA)-MRSA and that *agr* type III is more common in CA-MRSA [30], there have been few previous reports regarding the annual trends of *agr* types in bloodstream isolates. Therefore, the results of *agr* types in the present study also suggest a transition in the genotype of MRSA from HA-MRSA to CA-MRSA.

Strains identified as *agr* type II in this study showed a significantly higher proportion of VCM MIC of 2 or 4 μg/mL compared to strains of other *agr* types. It has been reported that strains of *agr* type II exhibit lower susceptibility to VCM compared to strains of other *agr* types [31,32,33]. In this study, among the five MRSA strains with VCM MIC of 2 μg/mL detected in the third period (2016), two strains were identified as SCC*mec* type II/*agr* type II, while three strains were identified as SCC*mec* type IV/*agr* type I. There have been previous reports of VCM-intermediate *S. aureus* or heterogeneous VCM-intermediate *S. aureus* with the SCC*mec* type IV/*agr* type I genotype [34,35,36], indicating the need for vigilance regarding the VCM susceptibility of MRSA strains harboring this genotype.

POT analysis has been reported to exhibit high discriminatory power comparable to PFGE, MLST analysis, and repetitive sequence-based PCR [37,38,39,40]. Furthermore, POT analysis has been applied in epidemiological studies of MRSA and for infection control within hospital wards [20,41,42,43].

In this study, the analysis of POT types revealed 128 epidemic clones among MRSA bloodstream isolates, defined as those with the same POT type detected in two or more strains. Among them, the top five POT types accounted for 46% of the isolates. Among the top five POT types, those with characteristics of CA-MRSA, represented by SCC*mec* type IV strains (POT types 106-247-33, 106-137-80, and 106-9-80), were prevalent not only in our institution but also in other medical facilities in Japan [20,41,42,44,45]. On the other hand, the SCC*mec* type II associated with the genotype of HA-MRSA, POT type 93-218-56, has not been reported in other facilities, suggesting that it may be unique to our institution. Therefore, the POT type associated with the genotype of HA-MRSA appears to be institution-specific, while the POT type associated with the genotype of CA-MRSA reflects the possibility of regional or national trends.

The detection rate of strains that did not exhibit epidemic expansion in POT analysis (i.e., sporadic clones consisting of a single strain) has increased significantly from 2016 to 2021 compared to the period from 2010 to 2015. This suggested that there has been a diversification of POT types among bloodstream isolates since 2016.

The strains in Cluster 1 were predominantly identified as CC5-SCC*mec* type II/*agr* type II, and they dominated as the major causative strains among MRSA bloodstream isolates in our hospital until 2015. This CC5-SCC*mec* type II/*agr* type II genetic type was previously a dominant strain in Japan [2,4]. The strains in Cluster 2 were identified as CC1-SCC*mec* type IV/*agr* type III and have been detected frequently since 2016. In addition, this cluster, with the exception of one strain, was identified as ST2725. CC1 MRSA bloodstream isolates have recently shown an increasing trend in Japan [17,46], with a particularly high prevalence of ST2725 reported in the Kyushu region [18]. Our analysis also suggested an increase in CC1 MRSA in recent years among bloodstream isolates. Cluster 3 strains were predominantly identified as CC8-ST8 and were detected throughout the study period. Interestingly, CC8-ST8 strains were mainly SCC*mec* type I/*agr* type I until 2015, but their prevalence decreased thereafter, with an increase in SCC*mec* type IV/*agr* type I, although the reason for this change remains unclear. While it has been reported that diverse SCC*mec* types exist within CC8-ST8 [47], to our knowledge, there have been no reports of a transition in SCC*mec* from type I to type IV within CC8-ST8 strains.

In this study, only one strain with the POT type 106-77-113, testing positive for PVL (*lukS-PV-lukF-PV*) and ACME (*arc*, *opp3*), was detected in 2020. It has been reported that many PVL-producing strains, including the possibility of USA300, exhibit the POT type 106-77-113 [48,49,50]. In recent years, the detection rate of PVL-positive strains, including USA300, has been increasing primarily in skin isolates within healthcare facilities [51]. However, the detection rate in blood isolates was reported to be very low [18,36,52], and our results were consistent with this observation.

This study had several limitations. First, this was a retrospective investigation of MRSA bloodstream isolates in a single facility. Second, the study focused exclusively on bloodstream isolates, which limited the ability to track nosocomial transmission. Third, the number of isolates subjected to MLST analysis in this study represented only a subset of epidemic strains. Fourth, we did not perform whole-genome sequencing for detailed analysis, such as the examination of single nucleotide polymorphisms, and therefore missed the opportunity to confirm hidden nosocomial transmissions [36]. However, within the scope of this study, only two instances were observed where strains with the same genotype were detected in the same ward within 1 month. Fifth, the investigation was conducted solely in a single facility, and due to insufficient information on patient backgrounds before transfer, accurate evaluation of epidemiological data was not possible. Nevertheless, this study revealed the long-term genetic trends of MRSA bloodstream isolates within the same healthcare facility in Japan. From a clinical perspective, the fact that there was a shift in MRSA bacteremia isolates in this study from hospital-specific strains that were endemic in hospitals to those that are endemic in the community suggests the importance of a broader public health approach in the prevention of MRSA bacteremia, looking not only at nosocomial infection control but also at the prevention of community-acquired infections in the community.

## 4. Materials and Methods

### 4.1. Clinical Isolates

This study was conducted at Fukuoka University Hospital, a university-affiliated, tertiary hospital in southwestern Japan. Over the 12-year period from 2010 to 2021, molecular epidemiological analysis was performed using 245 strains of MRSA isolated from the blood of hospitalized patients, with the exclusion of duplicate strains from the same individuals from a total of 281 strains. Isolates and the associated data were accessed between 1 October 2018 and 29 February 2024. To assess the longitudinal trends in MRSA, we divided this 12-year period into the first period (2010–2012), second period (2013–2015), third period (2016–2018), and fourth period (2019–2021). MRSA isolates were identified by standard phenotypic procedures and stored at −80 °C [53]. Prior to DNA extraction, they were cultivated at 37 °C with shaking in Difco™ M-*Staphylococcus* Broth (Becton, Dickinson and Company, Franklin Lakes, NJ, USA). Genomic DNA extraction was carried out using a Cica Geneus DNA Extraction Reagent Kit (Kanto Chemical, Tokyo, Japan) in accordance with the manufacturer’s recommended protocol. The obtained genomic DNA was utilized as a template for all analyses.

### 4.2. SCCmec Typing, Agr Typing, and Identification of Virulence Genes

SCC*mec* typing [54], *agr* typing [55], and the detection of PVL (*lukS-PV-lukF-PV*) [56] and ACME (*arc*, *opp3*) [57] were conducted using previously established methods. The ATCC BAA-1556 strain (USA300 clone) was used as a positive control for PVL and ACME detection.

### 4.3. POT Type Detection

POT analysis was conducted using a Cica Geneus^®^ Staph POT KIT (Kanto Chemical, Tokyo, Japan) [37,58]. POT analysis, a molecular epidemiological method with discriminatory power equivalent to pulsed-field gel electrophoresis (PFGE) [37,38,39], involves the detection of 22 phage-derived open reading frames (ORFs) effective for strain identification. Following the detection of these ORFs, the possession patterns were converted into numerical values, and the POT types (POT1-POT2-POT3) were determined. POT1 targets SCC*mec*, while POT2 and POT3 target phage-derived ORFs. In this study, strains in which the same POT type was detected in two or more isolates were classified as epidemic clones, whereas strains in which no common POT type was detected were considered to be sporadic clones.

### 4.4. MLST Analysis

MLST analysis was performed on a randomly selected subset of the epidemic clones identified based on POT types comprising a total of 92 strains [59]. Genotypes were classified into ST and clonal complexes (CC) following the MLST database (https://pubmlst.org).

### 4.5. Antimicrobial Resistance Profile

We investigated the drug susceptibility of a total of 244 strains, excluding one isolate for which information was not recorded in the medical records, for all drugs except DAP. As DAP was not used at our hospital from 2010 to 2012, there were no data on its MIC and drug sensitivity. Therefore, we investigated the drug susceptibility of a total of 179 isolates from 2013 for DAP.

Antimicrobial susceptibility testing was conducted using VITEK^®^2 (Sysmex bioMérieux, Tokyo, Japan). The antimicrobial agents investigated included GM, EM, CLDM, MINO, LVFX, FOM, ABK, TEIC, VCM, LZD, and DAP. Sensitivity to antimicrobial agents, excluding FOM, was determined according to the guidelines of the Clinical and Laboratory Standards Institute (CLSI) [60]. Sensitivity to FOM was determined following the guidelines of the European Committee on Antimicrobial Susceptibility Testing (EUCAST Version 10, 2020). The resistance breakpoints of the following antimicrobial agents were determined according to the CLSI: GM, ≥16 μg/mL; EM, ≥8 μg/mL; CLDM, ≥4 μg/mL; MINO, ≥16 μg/mL; LVFX, ≥4 μg/mL; ABK, ≥16 μg/mL; TEIC, ≥32 μg/mL; VCM, ≥16 μg/mL; LZD, ≥8 μg/mL; and DAP, not defined (N/D). As there was no defined breakpoint for ABK, the breakpoint for GM was used as a substitute. The resistance breakpoint for FOM was determined according to the guidelines provided by the EUCAST: FOM, >32 μg/mL.

### 4.6. Statistical Analysis

Differences in genotype detection rates were assessed using the χ^2^ test. For genotype clustering of MRSA bloodstream isolates, hierarchical clustering was performed using seven housekeeping gene numbers of MLST and POT1, 2, and 3 numbers. The analysis utilized the Ward method with Euclidean squared distance. All statistical analyses were conducted using EZR software version 1.62 (Jichi Medical University, Saitama Medical Center, Japan). In all analyses, *p* < 0.05 was taken to indicate statistical significance.

## 5. Conclusions

In conclusion, this study showed that the genotype of MRSA bloodstream isolates in our institution shifted from SCC*mec* type II/*agr* type II to SCC*mec* type IV/*agr* type III around 2016, with a notable increase in CC1 MRSA. The significant increase in sporadic isolates suggested the diversification of genotypes in recent years.

## Figures and Tables

**Table 1:** Annual distribution of the mode of MIC values and resistance rates to antimicrobial agents for 244 MRSA blood isolates.

Antimicrobial Drug ^a^	2010–2012 (*n* = 65)		2013–2015 (*n* = 59)		2016–2018 (*n* = 68)		2019–2021 (*n* = 52)		Total	
	Mode MIC [μg/mL] ^b^	R (%) ^c^	Mode MIC [μg/mL]	R (%)	Mode MIC [μg/mL]	R (%)	Mode MIC [μg/mL]	R (%)	Mode MIC [μg/mL]	R (%)
GM	≥16	57%	≥16	53%	≥16	51%	≤0.5	29%	≥16	48%
EM	≥8	83%	≥8	81%	≥8	65%	≥8	55%	≥8	72%
CLDM	≥8	62%	≥8	64%	≤0.25	32%	≤0.25	19%	≤0.25	45%
MINO	≥16	46%	≥16	53%	≤0.5	21%	≤0.5	15%	≤0.5	34%
LVFX	≥8	77%	≥8	85%	≥8	76%	≥8	75%	≥8	79%
FOM	≥8	23%	≥8	34%	≥8	12%	≥8	6%	≥8	19%
ABK	≤1	2%	≤1	0%	≤1	1%	≤1	0%	≤1	1%
TEIC	≤0.5	0%	≤0.5	0%	≤0.5	0%	≤0.5	0%	≤0.5	0%
VCM	1	0%	1	0%	1	0%	1	0%	1	0%
LZD	2	0%	2	0%	2	0%	2	0%	2	0%
DAP	N/D ^d^	N/D	1	N/D	0.25	N/D	0.25	N/D	0.25	N/D

^a^ ABK, arbekacin; CLDM, clindamycin; DAP, daptomycin; EM, erythromycin; FOM, fosfomycin; GM, gentamicin; LVFX, levofloxacin; LZD, linezolid; MINO, minocycline; TEIC, teicoplanin; VCM, vancomycin. ^b^ Mode MIC, the mode of MIC (μg/mL). ^c^ R, resistant. ^d^ N/D, not defined.

## Data Availability

All data are provided in the manuscript and Supplementary Information.

## Supplementary Materials

The following supporting information can be downloaded at: https://www.mdpi.com/article/10.3390/antibiotics13070670/s1, Table S1: Molecular epidemiological analysis and antimicrobial susceptibility testing results of MRSA blood isolates detected at Fukuoka University Hospital (245 isolates). The four periods were as follows: Period 1, 2010–2012; Period 2, 2013–2015; Period 3, 2016–2018; and Period 4, 2019–2021). N/T, non-typeable. N/A, not available. * Non-susceptibility to DAP.
